# Supplementary material for: Selection for growth drives the emergence of genetic heredity in protocells
Source: PLoS Biol. 2026 Mar 30;24(3):e3003544. doi: 10.1371/journal.pbio.3003544 (PMC13056260; doi:10.1371/journal.pbio.3003544)
Supplement: S1 Text — Detailed description of the model. (DOCX) [file pbio.3003544.s001.docx]

**Supplemental information to “First growth, then information: the path to genetic heredity in protocells”**

**Raquel Nunes Palmeira, Marco Colnaghi, Andrew Pomiankowski, Nick Lane**

Department of Genetics, Evolution and Environment, University College London, UK

## **S1 Text. Model structure**

## **S1 Text. Model structure**

### **I. Protocell content**

The populations of nucleotides and peptides are represented as matrices $N$and $A$ respectively (Figure S1). Each element of these matrices represents the number of nucleotide or peptide molecules with a certain length and number of hydrophobic monomers.

In matrix $N$, the position ($l, i$) concerns the number of nucleotide polymers of length $l$, of which $i$ are hydrophobic monomers (i.e. purines) and $l-i$ are hydrophilic monomers (i.e. pyrimidines). Because there cannot be a molecule of length 0 but it is possible to have molecule with 0 hydrophilic monomers in its composition, the value of $l$ starts at 1 while the value of $i$ starts at 0. For instance, $N_{1,0}=10$ means that there are 10 pyrimidines, $N_{1,1}=11$ means there are 11 purines, and $N_{3,2}=3$ means there are 3 polymers of length 3 which contain 2 purines and one pyrimidine (Figure S1). Likewise, in the peptide matrix, $P_{l,i}=X$ means that there are $X$ peptides of length $l$, containing $i$ hydrophobic and $l-i$ hydrophilic amino acid monomers. For simplicity, only two types of amino acid are considered. In this model, the sequence order of nucleotides or amino acids within a polymer is not considered, only the length and proportion of hydrophobic/hydrophilic monomers.


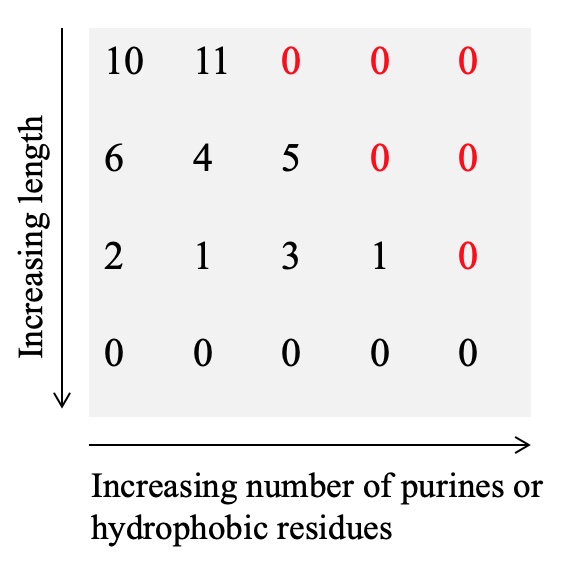


FS1 igure A An example of a population of molecules. The row number indicates the length of the molecule, while the column number indicates the number of hydrophobic monomers in each molecule. Zeroes in red are positions that cannot be filled. The same matrix structure relates to nucleotides (N) and peptides (A).

### **II. Protocell dynamics**

The equations in the section below provide average-value approximations of the stochastic, discrete-time simulation algorithm used. Each timestep corresponds to one ‘turn’ of the model (Fig.1), during which a fixed sequence of processes acts on the contents of each protocell.

#### Monomer addition

#### At each time point ($t$), carbon fixation adds $n$ nucleotide monomers to the protocell. $n$ is the result of sampling a binomial distribution with $n_{max}$ trials and probability $p_{n}$ (this probability can be increased by catalysis, see Catalysis section). Theseare split between a proportion $r$ that are hydrophilic and $1-r$ that are hydrophobic. The change in the number of monomers due to carbon fixation is

|  | ${\Delta N}_{1,0}^{cfix} =N_{1,0}\left( t \right)+n$, | (1a) |
| --- | --- | --- |

|  | ${\Delta N}_{1,1}^{cfix} =N_{1,1}\left( t \right)+n \left( 1-r \right)$. | (1b) |
| --- | --- | --- |

In a similar way, carbon fixation adds $m$ amino acid monomers, also sampled from a binomial distribution, this time with $m_{max}$ trials and probability $p_{m}$ that can be increased by catalysis. $m$ is also split also into hydrophilic and hydrophobic forms by the proportion $h$,

|  | ${\Delta P}_{1,0}^{cfix} =P_{1,0}\left( t \right)+mh$, | (2a) |
| --- | --- | --- |
|  | ${\Delta P}_{1,1}^{cfix} =P_{1,1}\left( t \right)+m\left( 1-h \right)$. | (2b) |
|  |  |  |

#### Random polymerisation of nucleotides

#### Random polymerisation (polymerisation of nucleotides without templates) occurs when a nucleotide monomer joins to another monomer or to an existing polymer. The probability of polymerisation is proportional to the numbers of the different molecules and is taken to be independent of the length or hydrophobicity of the molecules involved. To model this, each monomer in the system is randomly paired with another molecule and polymerisation occurs with probability $p_{p}.$ A pair $N\left( l,i \right)$ and $N\left( j,k \right)$ that polymerise increase the number of $N\left( l+j,i +k \right)$by one, and reduce the numbers of $N\left( l,i \right)$ and $N\left( j,k \right)$ by one. The average change in the number of polymers is

|  | $<{\Delta N}_{l,i}^{pol}\left( t+1 \right)> ={\frac{1}{2}N}_{total}\left( t \right)\left[ \frac{N_{l-1,i-1}\left( t \right)}{N_{total}\left( t \right)}\frac{N_{1,1}\left( t \right)}{N_{total}\left( t \right)} +\frac{N_{l-1,i}\left( t \right)}{N_{total}\left( t \right)}\frac{N_{1,0}\left( t \right)}{N_{total}\left( t \right)} - \left( \frac{N_{1,0}\left( t \right)}{N_{total}\left( t \right)}+\frac{N_{1,1}\left( t \right)}{N_{total}\left( t \right)} \right) \right]p_{p}$. | (3) |
| --- | --- | --- |
|  |  |  |

The first term in the equation is the formation of polymers $N_{l,i}$(length $l$, with purine content $i$) from the binding of a pyrimidine monomer, the second term is the formation of polymers $N_{l,i}$ from the binding a purine monomer, and the third term is the loss of the $N_{l,i}$ polymers that have gained either a purine or a pyrimidine, all terms are multiplied by approximate number of interacting pairs of molecules and the probability of random polymerisation.

The average change in the number of monomers due to polymerisation is

|  | $<{\Delta N}_{1,0}^{pol}\left( t+1 \right)> = - N_{1,0}\left( t \right) \frac{N_{1,0}\left( t \right)}{N\left( t \right)} \sum_{l>1,i=0}^{\infty} \frac{N_{l,i}\left( t \right)}{N\left( t \right)} p_{p}$ | (4a) |
| --- | --- | --- |

and

|  | $<\Delta N_{1,1}^{pol}\left( t+1 \right)> = - N_{1,1}\left( t \right) \frac{N_{1,1}\left( t \right)}{N\left( t \right)} \sum_{l>1,i=0}^{\infty} \frac{N_{l,i}\left( t \right)}{N\left( t \right)} p_{p}.$ | (4b) |
| --- | --- | --- |

#### Copying of nucleotide polymers

In addition, nucleotide polymers can arise from copying. Because copying occurs via base-pairing, the composition of the new polymer is reversed in relation to the template: for example, a template of $i$ purines and $l-i$ pyrimidines generates a new polymer with $l-i$ purines and $i$ pyrimidines. Each nucleotide polymer has probability $p_{c}$ per time step of being copied, and I assume this probability is independent of length or hydrophobicity.

The probability $p_{c}$ is used to obtain a matrix of templates to be copied. This is done through binomial sampling from the population matrix $N$ (for large samples and mid-ranged probabilities the normal approximation is used to improve computational efficiency).

|  | $<{\Delta N}_{l,l-i}^{copy}\left( t+1 \right)> = \sum_{i=0}^{l} N_{l,i}\left( t \right) p_{c}.$ | (5) |
| --- | --- | --- |

Incomplete copying is not considered, so copying will only occur if there are enough monomers to make up a complete copy.

Monomers used in copying are deducted from their respective positions. The average change in the number of monomeric purines due to copying is proportional to the total number of nucleotide templates containing pyrimidines times the number of pyrimidines in each template,

|  | $<{\Delta N}_{1,1}^{copy}\left( t+1 \right)> = - \sum_{l>1, j=0, i<l}^{\infty} N_{l,i}\left( t \right) p_{c}\left( l-i \right)$ | (6a) |
| --- | --- | --- |

and likewise for pyrimidine monomers,

|  | $<{\Delta N}_{1,0}^{copy}\left( t+1 \right)> = - \sum_{l>1, j=0, i>1}^{\infty} N_{l,i}\left( t \right) p_{c}i .$ | (6b) |
| --- | --- | --- |

#### Translation

In the model, peptides (amino acid polymers) arise from translation of nucleotide polymer templates. Here we assume that translation is based on hydrophobicity on a one-to-one relationship between nucleotides and amino acids. So, purines code for hydrophobic amino acids and pyrimidines code for hydrophilic amino acids. Each nucleotide polymer has a probability ($p_{t}$) per time step of serving as a template for translation. This is independent of the length and hydrophobicity of the polymer, but translation will only happen if there are enough amino acid monomers to make up the transcript.

Given a template nucleotide polymer in position $N\left( l,i \right)$, the translated peptide is added to position $P\left( l,i \right)$ of the amino acid matrix. The average change in the number of peptide polymers by translation is proportional to the number of nucleotide polymers of that length and the probability of translation:

|  | $<{\Delta A}_{l,i}^{trans}\left( t+1 \right)> = \sum_{i=0}^{l} N_{l,i}\left( t \right) p_{t}.$ | (7) |
| --- | --- | --- |

The monomers used to make the peptides are deducted from the matrix. The average change in the number of hydrophilic amino acids due to translation is proportional to:

|  | $<{\Delta A}_{1,0}^{trans}\left( t+1 \right)> =-\sum_{l>1,j=0,i<l}^{\infty} N_{l,i}\left( t \right) p_{t} \left( l-i \right),$ | (8a) |
| --- | --- | --- |

and likewise for hydrophobic monomers:

|  | $<{\Delta A}_{1,1}^{trans}\left( t+1 \right)> =-\sum_{l>1,j=0, i>1}^{\infty} N_{l,i}\left( t \right) p_{t} i.$ | (8b) |
| --- | --- | --- |

#### Polymer decay

Both nucleotide and peptide polymers decay by losing one monomer at a time. Each polymer has a probability ($p_{d}$) of losing a monomer per time step. Two scenarios were considered: probability of decay being independent of the length of the polymer, and the probability being weighted on the length of the polymer, such that the longer the polymer the more likely it would be to lose a monomer. The average change in the number of nucleotide polymers from decay is given by:

|  | $<{\Delta N}_{l,i}^{decay}\left( t+1 \right)> = p_{d}\left( - N_{l,i}\left( t \right)+N_{l+1,i+1}\left( t \right)\frac{i+1}{l+1}+N_{l+1,i}\left( t \right)\frac{l+1-i}{l+1} \right).$ | (9) |
| --- | --- | --- |

The first term is the loss of polymers in $N\left( l,i \right)$, the second and third terms the formation of $N\left( l,i \right)$ polymers from decay of longer polymer losing a purine or a pyrimidine. The knock-on change in the number of nucleotide monomers from decay is given by:

|  | $<{\Delta N}_{1,0}^{decay}\left( t+1 \right)> = p_{p} \sum_{l=2, i=0}^{\infty} N_{l,i} i$ | (10a) |
| --- | --- | --- |
|  | $<{\Delta N}_{1,1}^{decay}\left( t+1 \right)> = p_{p}\sum_{l=2, i=0}^{\infty} N_{l,i} \left( l-i \right)$ | (10b) |

Using the same logic, the average change in the number of amino acid polymers from decay is given by:

|  | $<{\Delta P}_{l,i}^{decay}\left( t+1 \right)> = p_{d}\left( - P_{l,i}\left( t \right)+P_{l+1,i+1}\left( t \right)\frac{i+1}{l+1}+P_{l+1,i}\left( t \right)\frac{l+1-i}{l+1} \right)$ | (11a) |
| --- | --- | --- |

|  | $<{\Delta P}_{1,0}^{decay}\left( t+1 \right)> = p_{p} \sum_{l=2, i=0}^{\infty} P_{l,i} i$ | (11b) |
| --- | --- | --- |
|  | $<{\Delta P}_{1,1}^{decay}\left( t+1 \right)> = p_{p}\sum_{l=2, i=0}^{\infty} P_{l,i} \left( l-i \right)$ | (11c) |

#### Catalysis

Peptides are assumed to be catalytic and can catalyse two processes: carbon fixation (monomer addition) and information function, which includes copying and translation (red arrows in Figure 2). The rate of catalysis of carbon fixation is denoted $k_{fix}$ and the rate of catalysis of information functions is denoted $k_{pol}$.

Catalysis is a function of the length and hydrophobicity of peptides, such that the longer the polymer the stronger its catalytic power, but this function plateaus at longer lengths. Catalysis is assumed to be optimum at specific relative hydrophobicities and modelled as a gaussian curve centred at a specific relative hydrophobicity. In the simpler version of this model, used for parameter sweeps, it is assumed hydrophobic peptides are better at catalysing carbon fixation as they tend to move to the membrane, and hydrophilic peptides are better at catalysing polymerisation as they tend to stay in the cytosol.

Catalysis of carbon fixation is modelled as an increase in the probabilities used to sample the number of monomers added at each time step ($p_{n}$ and $p_{m}$), by a proportion $k_{fix}$. Catalysis of templated polymerisation (copying and translation) is an increase in the probability of copying ($p_{c}$) and the probability of translation ($p_{t}$) by a proportion $k_{pol}$.

|  | $p_{n}^{cat}=p_{n}\left( 1+k_{fix} \right)$ | (12a) |
| --- | --- | --- |

|  | $p_{m}^{cat}=p_{m}\left( 1+k_{fix} \right)$ | (12b) |
| --- | --- | --- |

|  | $p_{c}^{cat}=p_{c}\left( 1+k_{pol} \right)$ | (12c) |
| --- | --- | --- |

|  | $p_{t}^{cat}=p_{t}\left( 1+k_{pol} \right)$ | (12d) |
| --- | --- | --- |

The catalytic constants $k_{fix}$ and $k_{pol}$ are the sum of the product of two functions (one is a function of length, and the other is a function of hydrophobicity) of all peptide polymers in the protocell.

|  | $k_{fix}= \sum_{l=2}^{inf} k_{fix}^{l}\left( l \right)k_{fix}^{h}\left( h \right)$ | (13a) |
| --- | --- | --- |

|  | $k_{pol}= \sum_{l=2}^{inf} k_{pol}^{l}\left( l \right)k_{pol}^{h}\left( h \right)$ | (13b) |
| --- | --- | --- |

$k_{fix}^{l}$ and $k_{pol}^{l}$ are functions of the length of a peptide, where $k_{fix max}^{l}$ $k_{pol max}^{l}$ are constants representing the value at which $k_{fix}^{l}$ and $k_{pol}^{l}$ plateau, and $\gamma_{fix}$ and $\gamma_{pol}$ are constants representing the length value when $k_{fix}^{l}$ is half of $k_{fix max}^{l}$, and $k_{pol}^{l}$ is half of $k_{pol max}^{l}$.

|  | $k_{fix}^{l}\left( l \right)\boldsymbol{=}\frac{1}{k_{fix max}^{l}\left( 1+e^{-l+\gamma_{c}} \right)}$ | (14a) |
| --- | --- | --- |

|  | $k_{pol}^{l}\left( l \right)\boldsymbol{=}\frac{1}{k_{pol max}^{l}\left( 1+e^{-l+\gamma_{p}} \right)}$ | (14b) |
| --- | --- | --- |


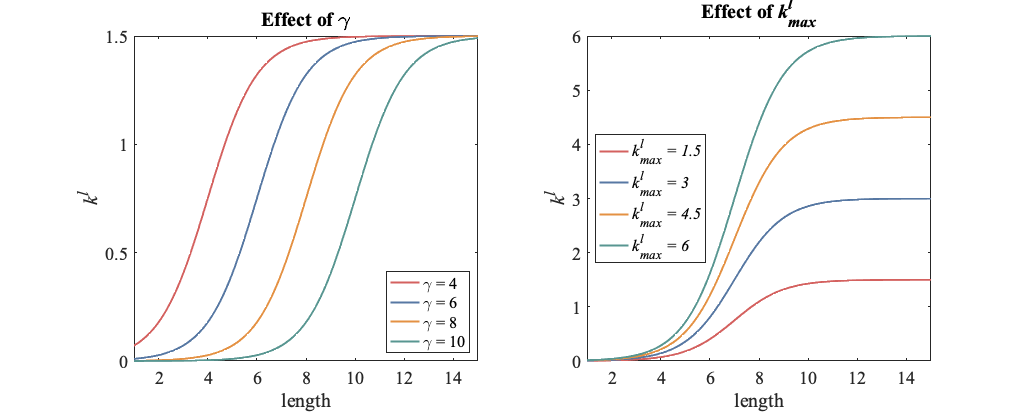


S1 Figure B. Effect of varying (a) $\boldsymbol{\gamma}$ and (b) $\boldsymbol{k}_{\boldsymbol{max}}^{\boldsymbol{l}}$ on the shape of the $\boldsymbol{k}^{\boldsymbol{l}}$ functions.

$k_{fix}^{h}$ and $k_{pol}^{h}$ are functions of the relative hydrophobicity of the peptides, where the two functions give gaussian curves centred around a specific relative hydrophobicity. Here, $\beta_{fix}$ and $\beta_{pol}$ are the relative hydrophobicities at the maximum point of $k_{fix}^{h}$ and $k_{pol}^{h}$. $\omega$ and $\upsilon$ are constants that give the steepness of the curve around the centre and the maximum value for $k_{fix}^{h}$ and $k_{pol}^{h}$, respectively.

|  | $k_{fix}^{h}= e^{\frac{{-(h-\beta_{fix})}^{2}}{\omega} \nu}$ | (16a) |
| --- | --- | --- |

|  | $k_{pol}^{h}= e^{\frac{{-(h-\beta_{pol})}^{2}}{\omega} \nu}$ | (16b) |
| --- | --- | --- |


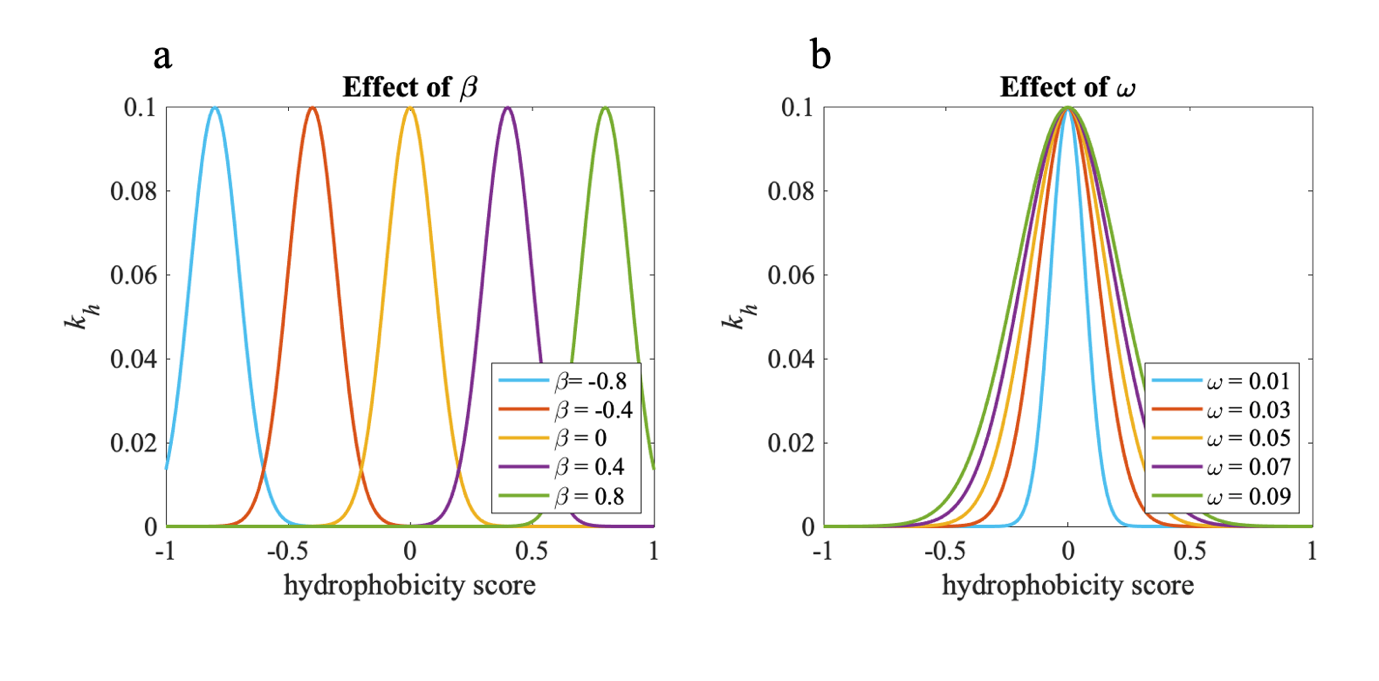


S1 Figure C. Effect of (a) varying $\boldsymbol{\beta}_{\boldsymbol{fix}}$ or $\boldsymbol{\beta}_{\boldsymbol{pol}}$ on the shape of the $\boldsymbol{k}^{\boldsymbol{h}}$ functions, and (b) varying $\boldsymbol{\omega}$ on the shape of the $\boldsymbol{k}^{\boldsymbol{h}}$ functions.

The simpler scenario, used for investigating the effect of individual parameters, considered hydrophobic peptides are better at catalysing carbon fixation as they tend to move to the membrane, and hydrophilic peptides are better at catalysing polymerisation as they tend to stay in the cytosol. In this scenario $k_{fix}^{h}$ and $k_{pol}^{h}$ are exponential functions of the hydrophobicity of a peptide polymer, where $\alpha_{fix}$ and $\alpha_{pol}$ are constants and $h$ is a relative hydrophobicity that ranges between -1 (completely hydrophilic) and 1 (completely hydrophobic). These two functions are symmetric with respect to hydrophobicity, such that copying the optimal sequence for carbon fixation catalysis would result in the optimal sequence for catalysis of copying and translation.

|  | $k_{fix}^{h}\left( h \right)=\frac{e^{\left( -\alpha_{fix} h \right)}}{\tau}$ | (15a) |
| --- | --- | --- |

|  | $k_{pol}^{h}\left( h \right)=\frac{e^{\left( \alpha_{pol} h \right)}}{\tau}$ | (15b) |
| --- | --- | --- |


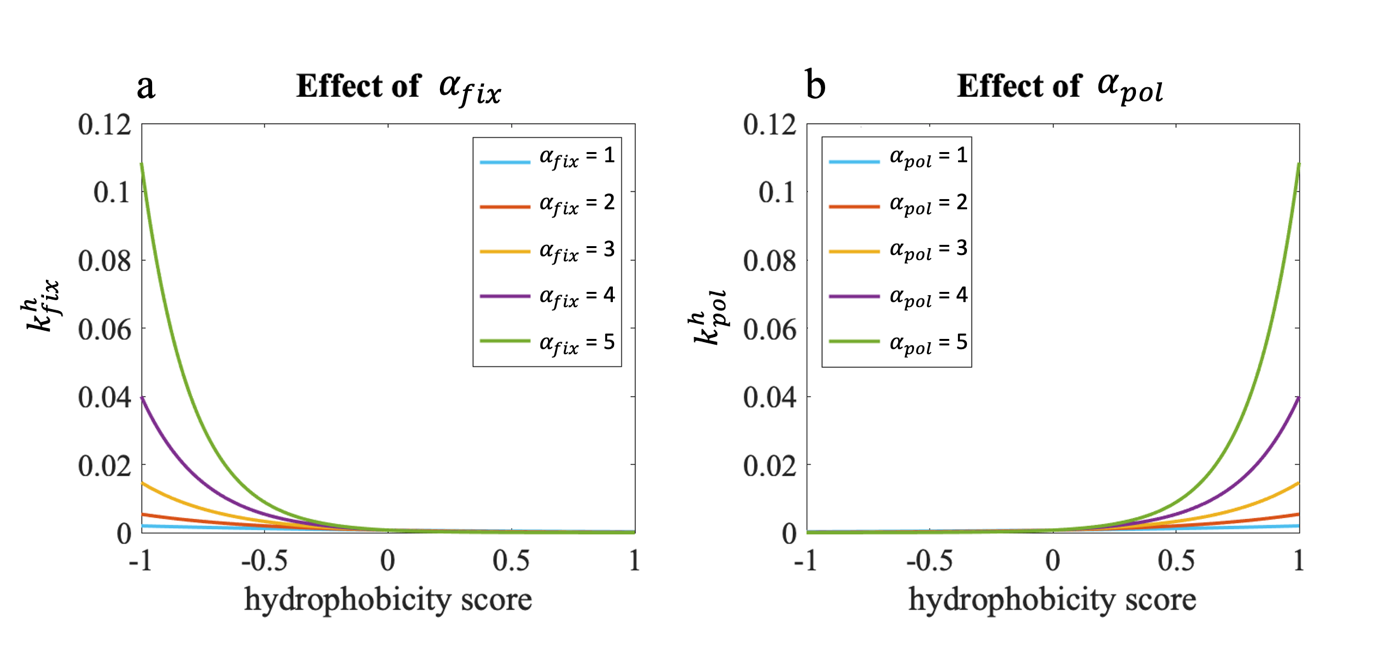


S1 Figure D. Effect of (a) varying $\boldsymbol{\alpha}_{\boldsymbol{fix}}$ on the shape of the $\boldsymbol{k}_{\boldsymbol{fix}}^{\boldsymbol{h}}$ function, and (b) varying $\boldsymbol{\alpha}_{\boldsymbol{pol}}$ on the shape of the $\boldsymbol{k}_{\boldsymbol{pol}}^{\boldsymbol{h}}$ function.

$h$ is calculated a function of hydrophobic residues in a peptide ($i$), the number of hydrophilic residues ($l-i$), and the total length ($l$).

|  | $h= \frac{i-\left( l-i \right)}{l}$ | (17) |
| --- | --- | --- |

#### Total change

Finally, the total change in the number of nucleotide molecules is given by:

|  | $<{\Delta N}_{i,j}^{total}\left( t+1 \right)> = <{\Delta N}_{i,j}^{cfix}\left( t+1 \right)>+<{\Delta N}_{i,j}^{pol}\left( t+1 \right)>+<{\Delta N}_{i,j}^{copy}\left( t+1 \right)>-<{\Delta N}_{i,j}^{decay}\left( t+1 \right)>$ | (18a) |
| --- | --- | --- |

And the total change in the number of peptide molecules is:

|  | $<{\Delta A}_{i,j}^{total}\left( t+1 \right)> = <{\Delta A}_{i,j}^{cfix}\left( t+1 \right)>+<{\Delta A}_{i,j}^{trans}\left( t+1 \right)>$  $- <{\Delta A}_{i,j}^{decay}\left( t+1 \right)>$ | (18b) |
| --- | --- | --- |

### **III. Modelling selection**

The evolution of a population of the protocells describe above was considered. This population was simulated for a set number of time steps “$t$” (where a time step is a “turn” of the model, as shown in Figure 1). At the end of each time step, if a protocell has reached a certain threshold size, it divides forming two daughter cells. Each daughter cell will contain a proportion of the contents of the mother cell. After every protocell division, one protocell is randomly removed from the population, this is done to maintain the population size constant for computational feasibility.

The size of a protocell is taken to be the total number of nucleotides in a cell, including both free monomers and those that are part of polymers. This is based on the assumption that the number of nucleotides is proportional to the number of fatty acids in the protocell, which are in turn, a proxy for membrane surface area.

To calculate the $N$ and $P$ matrices of the daughter cells after division, a proportion $x$ is independently sampled for each type of molecule in the mother protocell (i.e. every position in the $N$ and $P$ matrices) from a normal distribution truncated between 0 and 1, with mean 0.5 and standard deviation $s$. One daughter cell inherits an independent value $x$ of each type of molecule and the other daughter cell inherits $1 - x$.

### **IV. Measurements**

To look at the evolution of the protocells, the number of protocell divisions per time step and protocell distributions of nucleotides and amino acids were measured over the course of the simulations. The rate of protocell division was plotted as a moving sum to smooth out the curve and aid visualisation (e.g. Figure 3a). The distributions of nucleotide and peptide polymers were plotted as heatmaps, where every position holds the count for a polymer with a certain amount of hydrophobic and hydrophilic monomers (given by their positions in the x and y axes, respectively, e.g. Figure 3b-d).

Keeping track of the full distribution of nucleotides and amino acids for every time step requires excessive computational power. Instead, the distributions were only recorded for snapshots at intervals of 2000 time steps. In most cases only the snapshot distributions at t = 8000 were shown, as these reflect equilibrium distributions.
