# Supplementary material for: Selection for growth drives the emergence of genetic heredity in protocells
Source: PLoS Biol. 2026 Mar 30;24(3):e3003544. doi: 10.1371/journal.pbio.3003544 (PMC13056260; doi:10.1371/journal.pbio.3003544)
Supplement: S1 Fig — (a) Evolutionary change in the rate of protocell divisions (per 50 time steps) for low (pc= 0.001, blue lines) and high (pc= 0.1, orange lines) baseline probability of copying. Thin lines show individual populations and thick lines the mean. The mean distribution of nucleotides at three time points (t = 1,000, 4,000, and 8,000) shown by the red dotted lines, for (b–d) low (pc= 0.001) and (e and f) high (pc= 0.1) baseline probability of copying. Each square in the heatmap shows the log count of RNA molecules. The data and scripts used to generate this figure are available in the GitHub repository archived on Zenodo (https://doi.org/10.5281/zenodo.18940155, folder Figure S1). (DOCX) [file pbio.3003544.s002.docx]

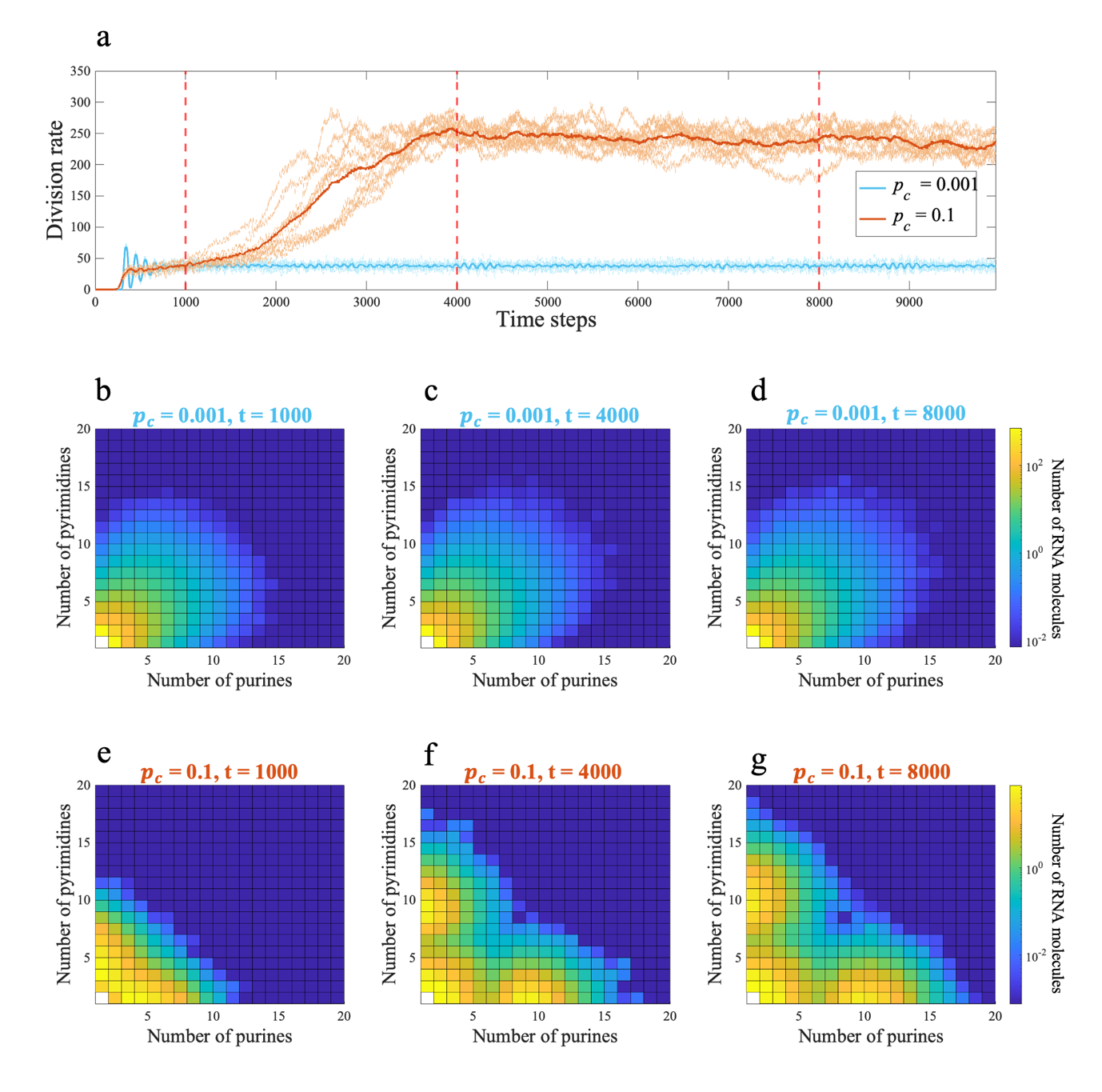


S1 Fig. Time-course of a model simulation. (a) Evolutionary change in the rate of protocell divisions (per 50 time steps) for low ($\boldsymbol{p}_{\boldsymbol{c}}\boldsymbol{= 0.001}$, blue lines) and high ($\boldsymbol{p}_{\boldsymbol{c}}\boldsymbol{= 0.1}$, orange lines) baseline probability of copying. Thin lines show individual populations and thick lines the mean. The mean distribution of nucleotides at three time points (t = 1000, 4000 and 8000) shown by the red dotted lines, for (b-d) low ($\boldsymbol{p}_{\boldsymbol{c}}\boldsymbol{= 0.001}$) and (e-f) high ($\boldsymbol{p}_{\boldsymbol{c}}\boldsymbol{= 0.1}$) baseline probability of copying. Each square in the heatmap shows the log count of RNA molecules composed of a specific numbers of purines and pyrimidines in the sequence, as indicated in the side bar. Parameters used in this simulation are shown in Table 1.
