# Supplementary material for: Selection for growth drives the emergence of genetic heredity in protocells
Source: PLoS Biol. 2026 Mar 30;24(3):e3003544. doi: 10.1371/journal.pbio.3003544 (PMC13056260; doi:10.1371/journal.pbio.3003544)
Supplement: S3 Fig — (a) Changes in the moving sum of protocell division in the population per time step are shown for pt = 0.0001 (blue), pt= 0.001(orange), and pt= 0.01 (yellow). (b–d) show the mean distribution of nucleotides across protocells at t = 8,000 (shown as a red dotted line in panel (a)) for (b) pt = 0.0001 (c) pt= 0.001, and (d) pt= 0.01. Each square in the heatmap shows the log count of a nucleotide molecule composed of a number of purines (its position in the x axis) and a number of pyrimidines (its position in the y axis). All other parameter values are in Table 1. The data and scripts used to generate this figure are available in the GitHub repository archived on Zenodo (https://doi.org/10.5281/zenodo.18940155, folder Figure S3). (DOCX) [file pbio.3003544.s004.docx]

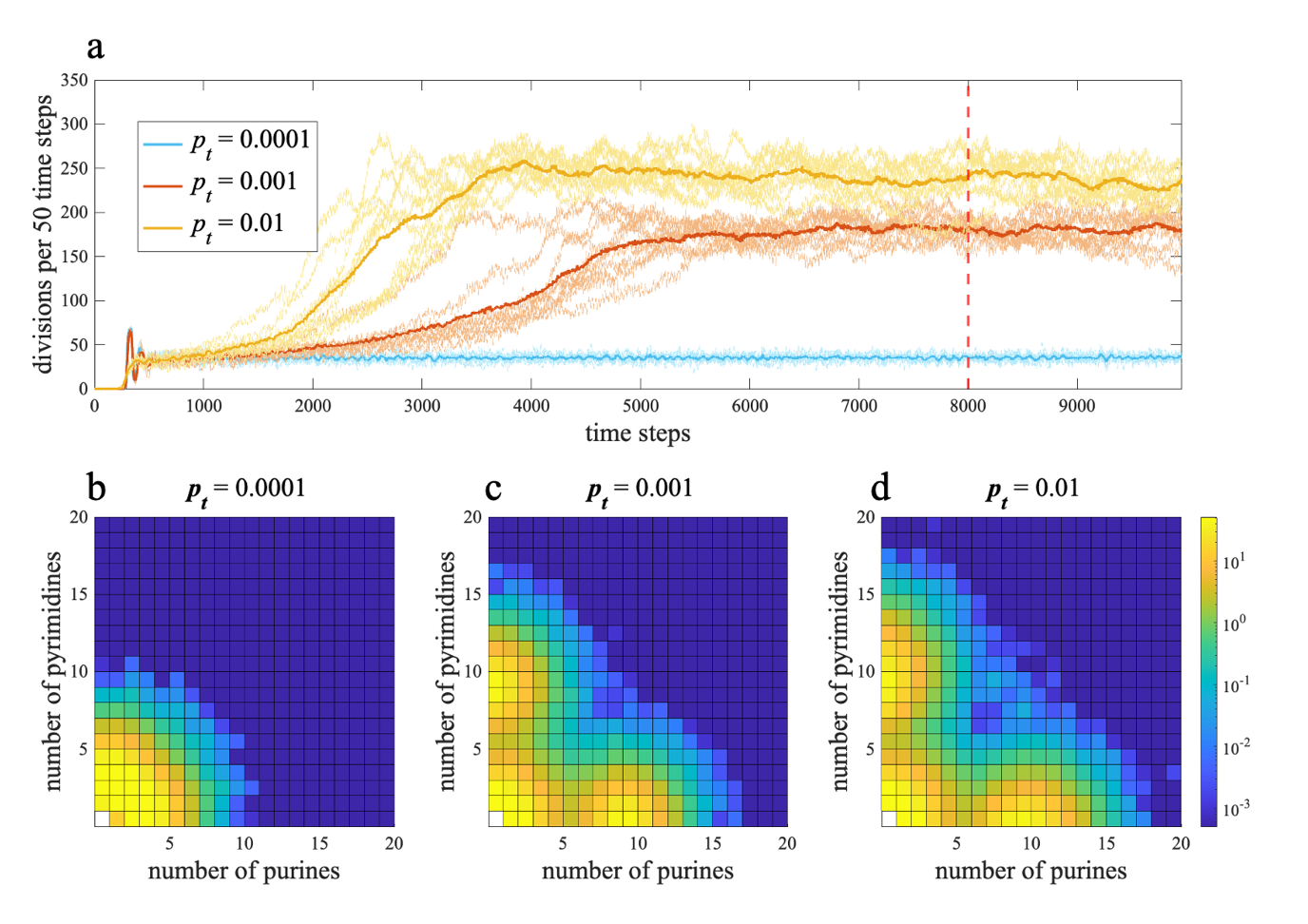


S3 Fig. Impact of probability of translation ($\boldsymbol{p}_{\boldsymbol{t}}$) on protocell division rate, composition and distributions of RNA in protocells. In (a) changes in the moving sum of protocell division in the population per time step are shown for $\boldsymbol{p}_{\boldsymbol{t}}$ = 0.0001 (blue), $\boldsymbol{p}_{\boldsymbol{t}}$= 0.001(orange) and $\boldsymbol{p}_{\boldsymbol{t}}$= 0.01 (yellow). (b) to (d) show the mean distribution of nucleotides across protocells at t = 8000 (shown as a red dotted line in panel (a)), for (b) $\boldsymbol{p}_{\boldsymbol{t}}$ = 0.0001, (c) $\boldsymbol{p}_{\boldsymbol{t}}$= 0.001 and (d) $\boldsymbol{p}_{\boldsymbol{t}}$= 0.01. Each square in the heatmap shows the log count of a nucleotide molecule composed of a number of purines (its position in the x axis) and a number of pyrimidines (its position in the y axis). All other parameter values are in Table 1.
