# Supplementary material for: Selection for growth drives the emergence of genetic heredity in protocells
Source: PLoS Biol. 2026 Mar 30;24(3):e3003544. doi: 10.1371/journal.pbio.3003544 (PMC13056260; doi:10.1371/journal.pbio.3003544)
Supplement: S4 Fig — (a) Catalytic rate (kl) as a function of peptide length (l) for increasing catalytic constants (γ=4 blue, γ=6 orange, γ=8 yellow, and γ=10 purple). (b) Evolutionary change in the rate of protocell divisions (per 50 time steps) for different values of γ. (c–f) show the mean Distribution of nucleotides across protocells at t=8000 for γ=4, (d) γ=6, (e) γ=8, and (f) γ=10. Each square in the heatmap shows the log count of RNA molecules composed of specific numbers of purines and pyrimidines. All other parameter values are as given in Table 1. The data and scripts used to generate this figure are available in the GitHub repository archived on Zenodo (https://doi.org/10.5281/zenodo.18940155, folder Figure S4). (DOCX) [file pbio.3003544.s005.docx]

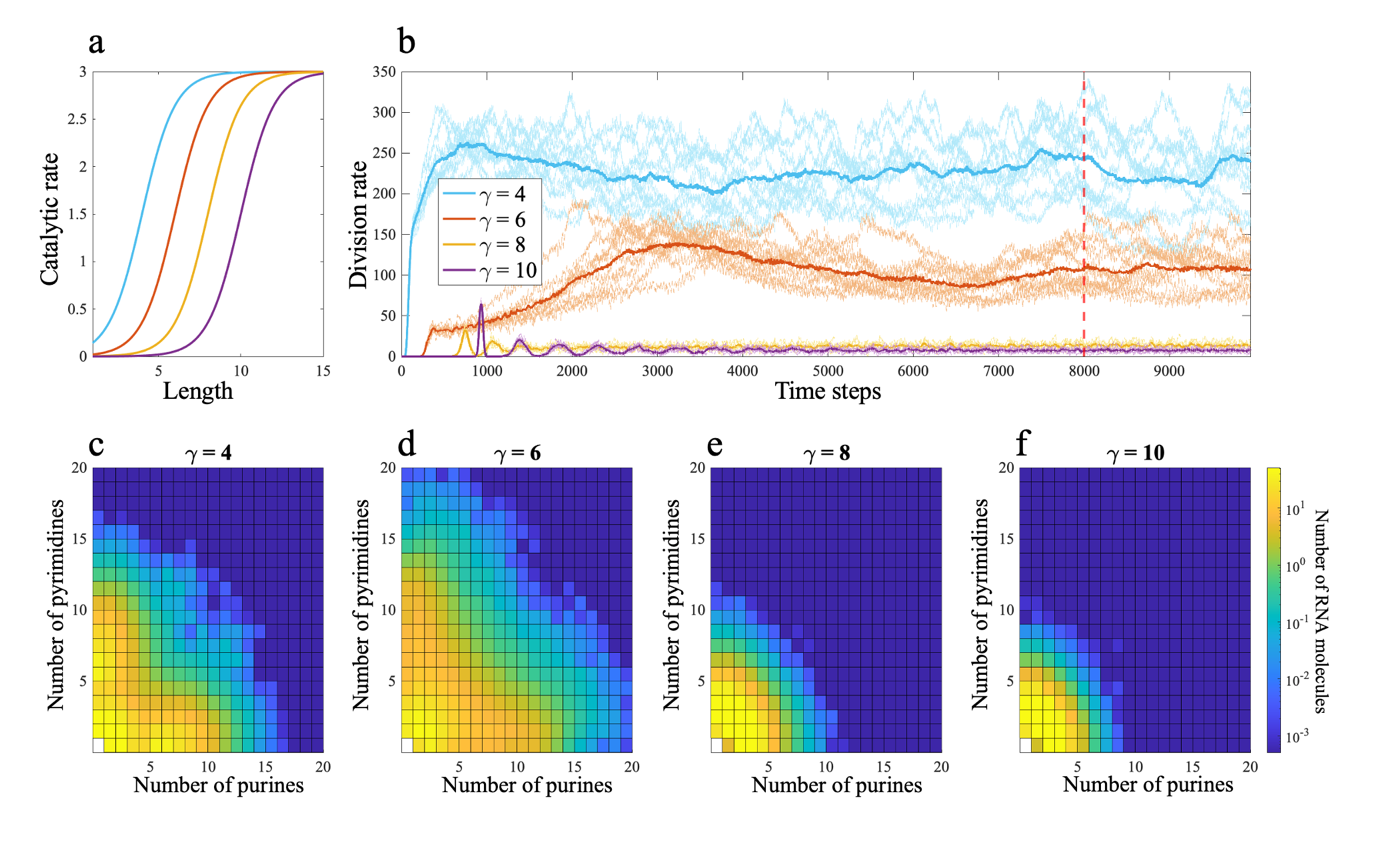


S4 Fig. Impact of varying the length of effective catalysts. (a) Catalytic rate ($\boldsymbol{k}_{\boldsymbol{l}}$) as a function of peptide length ($\boldsymbol{l}$) for increasing catalytic constants ($\boldsymbol{\gamma=4}$ blue, $\boldsymbol{\gamma=6}$ orange, $\boldsymbol{\gamma=8}$ yellow and $\boldsymbol{\gamma=10}$ purple). (b) Evolutionary change in the rate of protocell divisions (per 50 time steps) for different values of $\boldsymbol{\gamma}$. (c) to (f) show the mean distribution of nucleotides across protocells at $\boldsymbol{t=8000}$ (red line in panel (a)) for (c) $\boldsymbol{\gamma=4}$, (d) $\boldsymbol{\gamma=6}$, (e) $\boldsymbol{\gamma=8}$ and (f) $\boldsymbol{\gamma=10}$. Each square in the heatmap shows the log count of RNA molecules composed of specific numbers of purines and pyrimidines. All other parameter values are as given in Table 1.
