# Supplementary material for: Selection for growth drives the emergence of genetic heredity in protocells
Source: PLoS Biol. 2026 Mar 30;24(3):e3003544. doi: 10.1371/journal.pbio.3003544 (PMC13056260; doi:10.1371/journal.pbio.3003544)
Supplement: S5 Fig — Panel (a) shows the moving sum of protocell divisions over 50 time steps, and panels (b and c) show the average RNA compositions of protocells at time step 8,000. Catalytic rates (kl and kh) depend on the optimal catalytic length (γ) and hydrophobicity (β) for CO₂ fixation (γfix, βfix) and for templated polymerization (γpol, βpol). In panel (b) (blue line in panel a), γfix= 6, γpol= 8, βfix = 0.8, and βpol= –0.2. In panel (c) (orange line in panel a) γfix= 6, γpol = 8, βfix = 0.8, and βpol = –0.2. Each square in the heatmaps shows the log count of RNA molecules with a given number of purines and pyrimidines. All other parameters are listed in Table 1. The data and scripts used to generate this figure are available in the GitHub repository archived on Zenodo (https://doi.org/10.5281/zenodo.18940155, folder Figure S5). (DOCX) [file pbio.3003544.s006.docx]

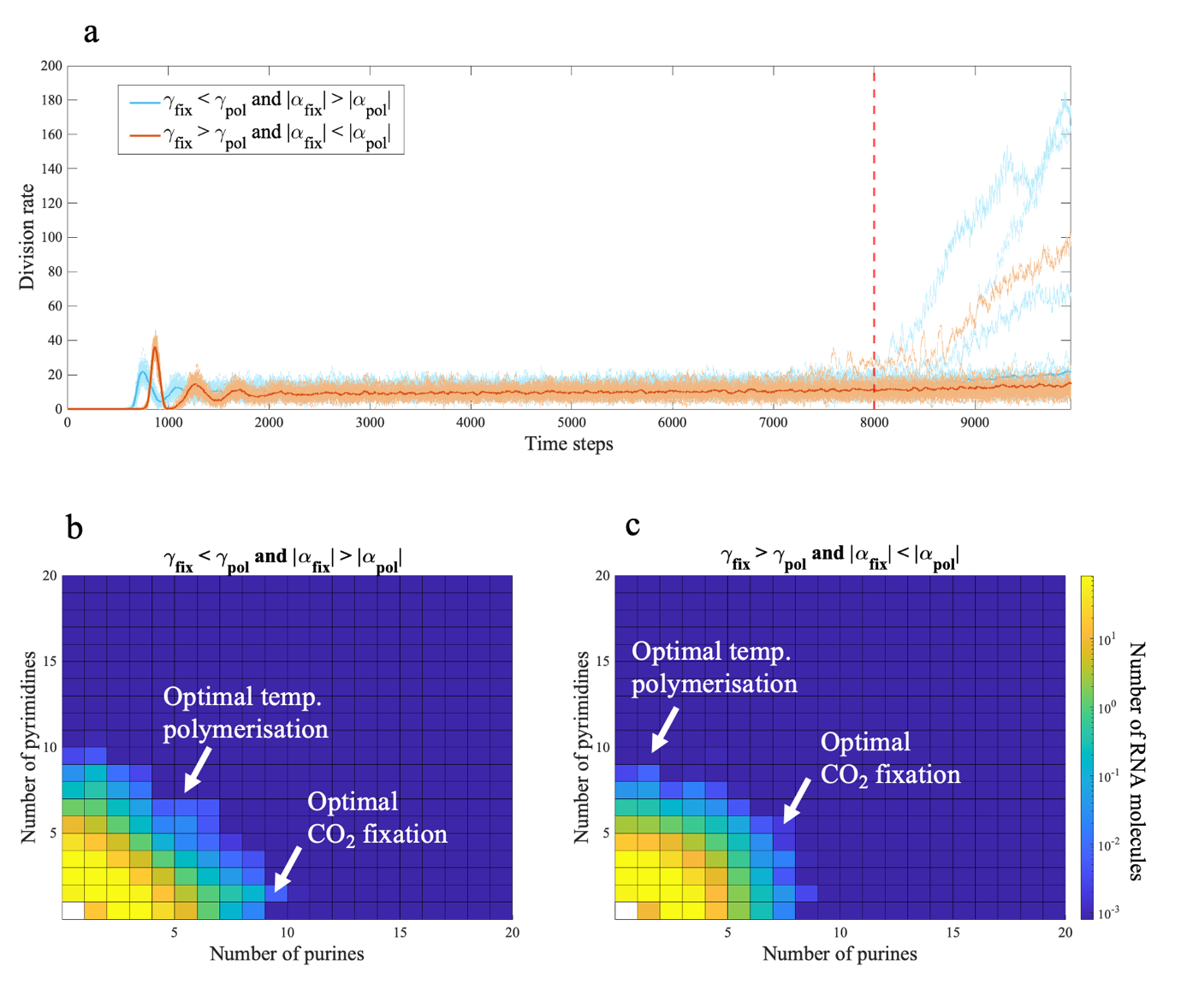


**S5 Fig. Evolution of protocells with different catalytic optima for CO₂ fixation and templated polymerisation.** Panel (a) shows the moving sum of protocell divisions over 50 time steps, and panels (b–c) show the average RNA compositions of protocells at time step 8000. Catalytic rates ($k_{l}$ and $k_{h}$) depend on the optimal catalytic length ($\gamma$) and hydrophobicity ($\beta$) for CO₂ fixation ($\gamma_{fix}$, $\beta_{fix}$) and for templated polymerisation ($\gamma_{pol}$, $\beta_{pol}$). In panel (b) (blue line in panel (a)), $\gamma_{fix}$= 6, $\gamma_{pol}$= 8, $\beta_{fix}$ = 0.8, and $\beta_{pol}$= –0.2. In panel (c) (orange line in panel (a)) $\gamma_{fix}$= 6, $\gamma_{pol}$ = 8, $\beta_{fix}$ = 0.8, and $\beta_{pol}$ = –0.2. Each square in the heatmaps shows the log count of RNA molecules with a given number of purines and pyrimidines. All other parameters are listed in Table 1.
