# Supplementary material for: Selection for growth drives the emergence of genetic heredity in protocells
Source: PLoS Biol. 2026 Mar 30;24(3):e3003544. doi: 10.1371/journal.pbio.3003544 (PMC13056260; doi:10.1371/journal.pbio.3003544)
Supplement: S6 Fig — This is investigated when CO2 fixation catalysts are easier to achieve at random (γfix= 6, γpol= 8, βfix = 0.2, and βpol= –0.8). (a) Evolutionary change in the rate of protocell divisions (per 50 time steps) for pp=0.01 (blue), pp=0.04 (orange), pp=0.08 (yellow), and pp=0.12 (purple). The mean distribution of nucleotides across protocells at t=8,000 (red dotted line in panel (a)) is shown for (b) pp=0.01, (c) pp=0.04, (d) pp=0.08, and (e) pp=0.12. Each square in the heatmap shows the log count of RNA molecules composed of specific numbers of purines and pyrimidines. All other parameter values are in Table 1. The data and scripts used to generate this figure are available in the GitHub repository archived on Zenodo (https://doi.org/10.5281/zenodo.18940155, folder Figure S6). (DOCX) [file pbio.3003544.s007.docx]

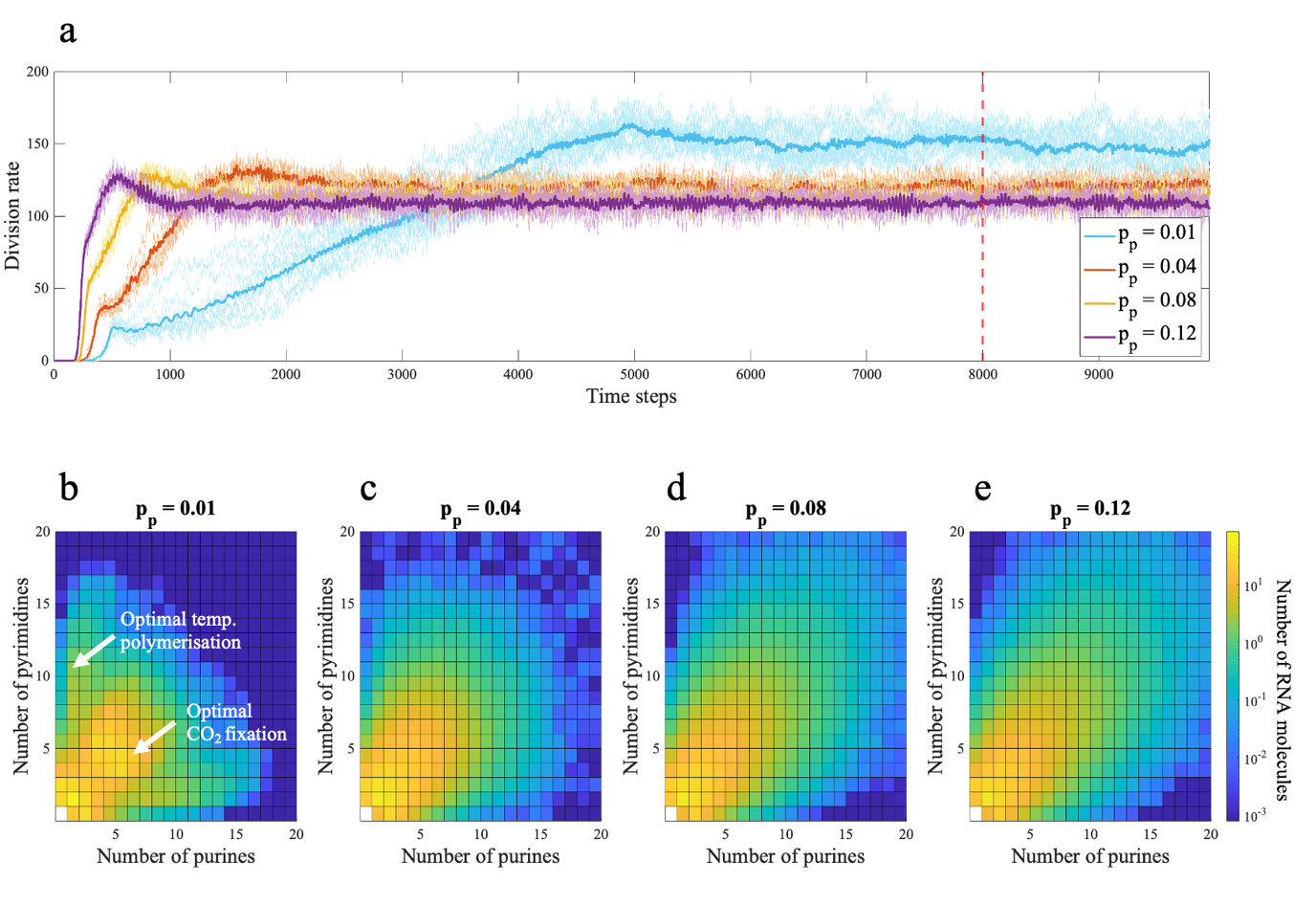


S6 Fig. The effect of varying the probability of random polymerisation ($\boldsymbol{p}_{\boldsymbol{p}}$). This is investigated when CO_2_ fixation catalysts are easier to achieve at random ($\boldsymbol{\gamma}_{\boldsymbol{fix}}$= 6, $\boldsymbol{\gamma}_{\boldsymbol{pol}}$= 8, $\boldsymbol{\beta}_{\boldsymbol{fix}}$ = 0.2, and $\boldsymbol{\beta}_{\boldsymbol{pol}}$= –0.8). (a) Evolutionary change in the rate of protocell divisions (per 50 time steps) for $\boldsymbol{p}_{\boldsymbol{p}}\boldsymbol{=0.01}$ (blue), $\boldsymbol{p}_{\boldsymbol{p}}\boldsymbol{=0.04}$ (orange), $\boldsymbol{p}_{\boldsymbol{p}}\boldsymbol{=0.08}$ (yellow) and $\boldsymbol{p}_{\boldsymbol{p}}\boldsymbol{=0.12}$ (purple). The mean distribution of nucleotides across protocells at $\boldsymbol{t=8,000}$ (red dotted line in panel (a)) is shown for (b) $\boldsymbol{p}_{\boldsymbol{p}}\boldsymbol{= 0.01}$, (c) $\boldsymbol{p}_{\boldsymbol{p}}\boldsymbol{= 0.04}$, (d) $\boldsymbol{p}_{\boldsymbol{p}}\boldsymbol{= 0.08}$ and (e) $\boldsymbol{p}_{\boldsymbol{p}}\boldsymbol{= 0.12}$. Each square in the heatmap shows the log count of RNA molecules composed of specific numbers of purines and pyrimidines. All other parameter values are in Table 1.
