# Supplementary material for: Selection for growth drives the emergence of genetic heredity in protocells
Source: PLoS Biol. 2026 Mar 30;24(3):e3003544. doi: 10.1371/journal.pbio.3003544 (PMC13056260; doi:10.1371/journal.pbio.3003544)
Supplement: Attachment — Submitted filename: Nunes Palmeira et al Response to Reviewers.docx [file pbio.3003544.s010.docx]

**Response to reviewers’ comments**

**Manuscript title:** First growth, then information: the path to genetic heredity in protocells

**Authors:** Raquel Nunes Palmeira, Marco Colnaghi, Andrew Pomiankowski, Nick Lane

We thank the editor and reviewers for their careful reading of our manuscript and for their constructive comments. We have revised the manuscript accordingly. Below, we respond to each comment. Reviewer comments are reproduced verbatim in italics. Our responses are in regular font in red. Changes to the manuscript and SI are indicated in red in the revised versions.

**Reviewer #1:**

*"First growth, then information: the path to genetic heredity in protocells" by Palmeira et al presents an exceptionally simple model for the evolution of metabolism, which is equated to protocell growth, and evolution of templated synthesis (here, equivalent to heredity) at the stage of(pre)life evolution.*

*The simplicity of the model is quite striking: hydrophobic peptides are assumed to be responsible growth (CO2 fixation) whereas hydrophilic ones are assigned to templated synthesis. The outcome of the simulations the authors perform under this model is quite obvious: for the protocells to reach a high division rate, the cost of growth has to be lower than the cost of templated synthesis. Hence 'First growth, then information'. This conclusion holds for autotrophic protocells, that is, in the absence of influx of monomers from the environment. In the heterotrophic version of the model, evolution of metabolism can be uncoupled from the evolution of information transmission unless the external supply of monomers is rate-liming.*

*This work is quite easy to read and understand but somewhat difficult to evaluate. On the one hand, the results are so intuitive that they potentially might be considered trivial. Indeed, how can replication and translation be possibly sustained if there is not steady, sufficient supply of monomers and energy...*

We thank the reviewer for their positive assessment of the clarity and accessibility of the manuscript. We agree the model is simple, deliberately so, and that its conclusions are intuitive at a broad level.

The main result is not simply that replication and translation require a steady supply of monomers. Rather, the model reveals that when functions enhancing copying arise more easily, they amplify existing sequences, which draws monomers away from exploration of sequence space. This suppresses the emergence of growth-enhancing functions that would increase monomer supply and enable sustained selection at the protocell level.

In contrast, when growth-enhancing functions evolve more easily, increased monomer production promotes sequence exploration and the evolution and retention of information-enhancing functions. This feedback between autotrophic growth and evolvability is more subtle than implied by R1, and has not been explored or demonstrated in previous models. We have tried to make this clearer in the revised MS (lines 306-308).

*However, exactly because of its simplicity, this work might become impactful in the origin of life field where 'metabolism first vs replication first' is still viewed as a serious conundrum. Hence my suggestion to the authors is to present this dilemma explicitly from the start, to set up the context for the modeling approach. This way, the work may be considered as a step towards the solution of an important problem in the origin of life research, and as such could be of interest to many.*

We agree that our paper speaks to the broader metabolism versus information-first debate. We have now made this connection explicit in the Introduction (lines 101 to 106). We clarify that, because the maintenance of information requires compartmentalisation, our model specifically addresses a stage in which protocells already possess rudimentary metabolism and heritable sequences, but the first selectable functions have not yet emerged. In this context, our results are directly relevant to the broader dilemma of metabolism vs. information-first by identifying the conditions under which growth-enhancing functions must precede information-enhancing ones.

We thank R1 again for their positive comments and agree these changes to the Introduction improve the paper.

**Reviewer #2:**

*In "First growth, then information: the path to genetic heredity in protocells" the authors develop and use a mathematical model to understand how RNA polymers in protocells could evolve longer coding sequences. Motivating this work is Eigen's paradox in which error-prone copying of sequences needs error-proofing sequences, but these are so long that they themselves would also need error-proofing sequences. While other models have addressed issues of preserving sequence information the authors raise questions concerning the purposes/functions of early sequences. The authors address these questions by considering a scenario in which growth and information preservation are coupled. Specifically, their model considers two essential processes affecting polymer production: the fidelity of sequence replication (information) and the production of monomers for sequence construction (growth). They study scenarios in which growth or information processes are easier to evolve by random chance. Importantly selection acts at the level of the protocell instead of just the sequences. Through their analyses they find that when sequences promoting growth are easier to evolve it leads to both growth and information-preserving sequences. Instead when sequences promoting information fidelity are easier to evolve, it stymies the population from evolving longer sequences. Overall, I enjoyed this paper and think its results in terms of concept and significance are suitable for publication. I have some questions concerning the modeling that should be addressed.*

We thank R2 for this excellent encapsulation of our paper.

*In terms of the modeling, many of the assumptions regarding the formulation of the model were well-justified. Certainly one could have done a more explicit model that took into account the actual sequences of the polymers, but that would likely introduce many additional parameters and complexity without fundamentally changing the main result. That said, I did have issues/questions with the modeling that I would like the authors to address.

First, in equation 3 in the Supplement there is an equation describing the average change in the number of polymers of a certain length and hydrophobocity. In this equation, binding occurs based on a certain probability and the product of three terms: 1. the number of a type of polymer and 2,3. the fractions of two types of polymers in the current population. This formulation seems a bit unusual. For one, scaling the terms by the current population of nucleotides is a different normalization than typically used. In mass-action models the normalization is often done by volume so that the variables are concentrations. If the volume is constant then this would mean that the binding probability term would just have different units. Instead, the authors scale by the population size, which affects the kinetics. It is effectively sampling from the existing polymer pool (with frequency-dependence) rather than using mass-action kinetics in a fixed volume.*

The normalisation used in Eq. 3 corresponds to frequency-dependent sampling from the existing molecular pool, which differs from a standard mass-action formulation. The equation approximates behaviour as a discrete-time model in which each timestep represents a ‘turn’ of the protocell dynamics (Fig. 1). During each step, molecules in a well-mixed protocell are randomly paired and polymerisation occurs with probability *p_p_*. This formulation captures the relative probabilities of production of each polymer type per time step (reflecting the accumulation and transmission of rudimentary ‘information’ at the protocell level) rather than detailed reaction kinetics, which would be essentially arbitrary. This modelling choice reflects the fact that protocells don’t have a constant volume - they grow and divide. The quantities that persist across generations and are visible to selection are relative abundances rather than concentrations. To make the meaning of the equations clearer to the reader, we’ve added a brief explanation, lines 36-38 of the SI.

*It is also not clear to me why the N_{l-1,i-1} term appears twice. To put it more clearly, suppose there were two types of molecules that might interact to give rise to a third, e.g. X+Y --p--> Z. A differential equations model of this might look something like dZ/dt= p[X][Y]. Yet, adopting the approach in this paper, the model would look something like dZ/dt=p[X] [X]/[X+Y+Z] [Y]/[X+Y+Z]. Perhaps the authors are making some assumptions that led to this model choice but from reading the text it is not obvious to me what they are.*

We thank the reviewer for noticing this. The repetition of the *N_l-1,i-1_* term in Eq. 3 is an error and has been corrected. It was not an error in the simulation algorithm, so the results are correct.

In the model itself, polymerisation occurs through random pairing of molecules within each timestep: approximately half the molecules in the protocell form interacting pairs, and the expected number of polymerisation events is given by the number of pairs multiplied by the proportions of the interacting molecular types and the probability of random polymerisation (*p_p_*). As in the original equation, there are three contributions to the average change in the number of polymers of type *N_l,i_*: (1) gain through interaction between *N_l-1,i-1_* and a purine monomer, (2) gain through interactions between *N_l-1,i_* and a pyrimidine monomer, and (3) loss of *N_l,i_* polymers that grow out of this class by acquiring a monomer of either type. Eq. 3 has been amended accordingly to provide a clearer expected-value approximation of the random-pairing algorithm used in the simulation. A sentence has been added after the amended equation to clarify the meaning of the first term (lines 60-61 of the SI).

*Second, disregarding incomplete RNA copies removes a potentially important source of shorter sequences that could possibly outcompete longer molecules. Including such sequence fragments could alter the balance between sequence construction and decay and thus change the steady state distribution.*

*Similarly, there is an assumption that all polymers have a probability "pd" of losing a monomer, which is weighted by length. Yet if a longer polymer has a higher chance of losing a monomer then it seems losing a middle monomer would generate two smaller polymer sequences instead of a monomer and polymer. I believe this could be fixed by simply assuming that monomer loss can only occur at the ends, though I don't know if there is any empirical motivation for this assumption.*

Thank you for these comments. We agree these are important assumptions. We’ve added some text about them and their repercussions in the Discussion, but we don’t believe these considerations change the qualitative results of the paper (lines 562-566 and 570-580).

Incomplete copies would increase the abundance of shorter polymers, and so reduce the fraction of polymers reaching functional length. This effect primarily shifts the quantitative boundary at which functional polymers become common, but does not change the qualitative ordering of evolutionary processes we identify in the paper. In the model, we set length and hydrophobicity as optimal for functional sequences enhancing copying and growth, but the precise locations of these thresholds are arbitrary, and we vary them. The central result is that evolving informational functions first hinders evolvability by diverting monomers into polymers that are not immediately beneficial at the protocell level, whereas evolving growth-promoting function first allows broader exploration of polymer space, which facilitates the eventual emergence and selective retention of copying functions. Introducing incomplete copies would bias the system towards shorter polymers and make functional sequences harder to attain, but does not alter this underlying logic.

Polymer decay is implemented in the model as the loss of monomers from the ends of polymers, with a probability that increases with polymer length. Allowing internal breakage would generate two shorter polymers rather than a polymer and a free monomer. This would increase the abundance of shorter polymers and effectively make monomers more limiting. This would put an even greater onus on the need for growth first, as it exacerbates the shortage of monomers. Hence our position is in fact quite conservative. As in the case of incomplete copying, this modification would shift quantitative boundaries but is unlikely to change the qualitative ordering of evolutionary outcomes identified by the model.

In fact there is also an empirical motivation for polymers to primarily lose monomers from the 3' end, as bacterial exonucleases such as PNPase and RNase initiate RNA degradation from the 3'🡪5' end (which is also the case for tRNA maturation and RNA processing more generally). This 3'🡪5' decay is not true of prebiotic chemistry (where attack from the 2' hydroxyl occurs anywhere along the chain), but degradation may still occur from the 3' end if it is not buried in a folded RNA. Given that the model concerns nascent functions, we could imagine that some small polymers might exert limited exonuclease activity from the 3' end. We have added a note to this effect in the revised paper on lines 570-580.

*Finally, most of the details of the model were in the Supplement. While I understand why this choice was made, it was challenging to understand how exactly the simulation worked. Perhaps there is computer code in some public repository?*

The code is available in a public github repository, which was indicated in the title page of the manuscript (<https://github.com/raquelnpalmeira/first_growth_then_information>). To make this more accessible to the reader we’ve added a sentence at the end of the methods section indicating that the full code for the model simulations is in the repository (lines 286-289). Apologies that this was not clearer for you.

*Reading the main text and the supplement, I think I have some sense of what is happening but I am unsure if I could precisely replicate their model. More clarification is needed, especially in the main text. Such clarification  is important because the results of the paper rely on these simulations. The authors have a schematic for the main processes in the protocell in Figure 1, but I think some more information would help clarify the structure of the model. For example, it would be useful to indicate that there is a population of protocells undergoing evolution via a Moran process. Also, it would be useful to depict how cell reproduction occurs, i.e. when the fatty acid population exceeds some threshold.  Last, it would be helpful to indicate the various sources and sinks for monomers---especially because these are manipulated in subsequent figures.*

We thank Reviewer 2 for their thorough and thoughtful assessment of our manuscript. We agree that it is important for the model to be easily reproducible by readers who may wish to explore or extend it.

To address this, we have added a clarifying paragraph at the beginning of the Model overview section in the main text (lines 152–157), explicitly stating that the model is an individual-based, stochastic model in discrete time, in which the processes described are implemented as probabilistic events. We have also revised the legend of Figure 1 to make clear that the diagram represents the internal dynamics of a single protocell embedded within a population, how population-level dynamics are implemented (via a Moran-type process), and which processes act as sources and sinks for monomers.

Finally, to further improve reproducibility, we have edited the README file in the public code repository so that it aligns more closely with the description in the main text and provides clearer guidance on how the code is structured and how simulations are run.

*Minor points:*

*The figure captions could benefit from some sentences describing the central result that should be observed. When reading this paper, I had to keep alternating between figure and sections in the Results to understand what I should be noting.*

We have added description of the main results in each figure caption.

*In S1.2 I think there is a typo in "with m_{max}ials "*

Thanks. We have corrected this typo in line 46 of the SI.
